# A Transcriptional Sequencing Analysis of Islet Stellate Cell and Pancreatic Stellate Cell

**DOI:** 10.1155/2018/7361684

**Published:** 2018-01-24

**Authors:** Xiaohang Wang, Wei Li, Juan Chen, Sheng Zhao, Shanhu Qiu, Han Yin, Vladmir Carvalho, Yunting Zhou, Ruifeng Shi, Jiannan Hu, Shenyi Li, Munire Nijiati, Zilin Sun

**Affiliations:** ^1^Department of Endocrinology, Institute of Diabetes, School of Medicine, Zhongda Hospital, Southeast University, Nanjing, China; ^2^Department of Biochemistry and Molecular Biology, School of Medicine, Southeast University, Nanjing, China

## Abstract

**Background:**

Our previous studies have shown that islet stellate cell (ISC), similar to pancreatic stellate cell (PSC) in phenotype and biological characters, may be responsible for the islet fibrosis in type 2 diabetes. To further identify the differences between PSC and ISC and for better understanding of the physiological function of ISC, we employed genome-wide transcriptional analysis on the PSCs and ISCs of Wistar rats.

**Method:**

PSCs and ISCs from each rat were primarily cultured at the same condition. Genome-wide transcriptional sequence of stellate cells was generated. The identified differentially expressed genes were validated using RT-PCR.

**Results:**

32 significant differentially expressed genes between PSCs and ISCs were identified. Moreover, collagen type 11a1 (*COL11A1*), was found to be expressed 2.91-fold higher in ISCs compared with PSCs, indicating that COL11A1 might be a potential key gene modulating the differences between PSC and ISC.

**Conclusions:**

Our study identified and validated the differences between PSC and ISC in genome-wide transcriptional scale, confirming the assumption that ISC and PSC are similar other than identical. Moreover, our data might be instrumental for further investigation of ISC and islet fibrosis, and some differential expressed genes may provide an insight into new therapeutic targets for type 2 diabetes.

## 1. Introduction

Type 2 diabetes mellitus (T2DM) is a chronic disorder which is characterized by *β*-cell dysfunction and insulin resistance [1, 2]. As the prevalence of type 2 diabetes continues to increase, it is imperative to seek a better understanding of its pathogenesis and find more efficient treatments to decrease the morbidity and ease the burden on the healthcare systems. Pancreatic stellate cells (PSCs) have been shown to play an important role in the pathogenesis of fibrosis in chronic pancreatitis and pancreatic cancer [3].

In a recent study, we identified a population of stellate cells growing outward of isolated islets which are similar, but not identical to classical PSCs, during the culture of islets from Wistar and Goto-Kakizaki (GK) rats. These cells are named islet stellate cells (ISCs) [4, 5], which have been verified to play important roles in islet fibrosis that promotes T2DM progression.

In normal conditions, PSCs stay quiescent and contain vitamin A-storing lipid droplets in their cytoplasm. When suffering from chronic inflammation or oxidative stress, PSCs will be activated and turned into myofibroblast cells, losing their vitamin A-storing lipid droplets, expressing *α*-SMA, ECM components, vimentin, and GFAP, producing cytokines and chemokines such as IL6, IL8, and monocyte chemoattractant protein- (MCP-) 1, as well as having higher proliferation and migration activities. Many recent studies have also shown that there are *α*-SMA-positive cells, identified as PSCs, in the models of islet fibrosis both in humans and animals with T2DM [6–9].

In the previous study, we compared Wistar rat's PSCs with ISCs and found that ISCs contained fewer vitamin A-storing lipid droplets and were more rapidly activated than PSCs in vitro. Activated ISCs express *α*-SMA, ECM components, vimentin, and GFAP, which is similar to activated PSCs. But ISCs have lower rates of proliferation and migration than PSCs in vitro, suggesting that ISCs are similar but not identical to PSCs in morphology and phenotype. In this study, we performed RNA-seq and real-time PCR validation on cultured Wistar rat's PSCs and ISCs to determine the gene differences and splicing variations and provided a global transcriptome comparison between ISCs and PSCs.

## 2. Materials and Methods

### 2.1. Animals

Three 10-week-old healthy male Wistar rats weighted 300–350 g were chosen for this study, yielding 3 biological duplicates. After measuring fasting blood glucose, the rats were given anesthesia and sacrificed to collect their pancreas. These rats were numbered A, B, and C, and PSCs from A, B, and C were named A1, B1, and C1, respectively. Similarly, the ISCs were named A12, B12, and C12, respectively. Housing and animal experiments were approved by Southeast University Animal Care and Use Committee according to institutional guidelines and national animal welfare.

### 2.2. Isolation and Culture of ISCs

Islets were isolated from pancreas as previously described [10]. Briefly, pancreas tissues were digested with collagenase V (1 mg/mL, *w/v*) (Sigma, St. Louis, MO, USA) at 37°C for 15 to 18 min. Islets were purified by handpicking twice under a stereomicroscope. Then, islets were precultured in RPMI-1640 supplemented with L-glutamine containing 10% (*v*/*v*) fetal bovine serum (FBS) (Invitrogen, Carlsbad, CA, USA) overnight, followed by handpicking.

After 48 h in culture, ISCs began to grow out of Wistar islets. Five days later, cells were subcultured in DMEM/Ham's F12 (1 : 1, *v*/*v*) containing 10% FBS. Then, cells at passages 3 were used for experiments.

### 2.3. Isolation and Culture of PSCs

PSCs were isolated from Wistar rats as described previously [11]. PSCs were cultured in Dulbecco's modified Eagle's medium (DMEM)/Ham's F12 (1 : 1, *v*/*v*) containing 10% fetal bovine serum (FBS) (Invitrogen, Carlsbad, USA). The cells were cultured at the same condition with ISCs. Cells at passages 3 were used for experiments.

### 2.4. Identification of ISCs via Immunofluorescent Assay

Cells were fixed in 4% paraformaldehyde in PBS for 20 min at room temperature. Immunofluorescent staining of stellate cells for *α*-SMA was performed. Cells were incubated overnight at 4°C with primary antibody (Abcam, 1 : 200), followed by a 1 h treatment with secondary antibody (Jackson ImmunoResearch Laboratories, 1 : 100). The sections in negative control group were incubated with PBS, instead of primary antibody. And the results indicated that the antibody and the staining worked well. Morphometric analyses were performed using Image J software.

### 2.5. mRNA Library Construction and Sequencing

Using the Trizol reagent (Invitrogen, CA, USA) according to the manufacturer's protocol, the total RNA was extracted. The purity and quantity of the total RNA were detected using RNA 6000 Nano LabChip Kit (Agilent, CA, USA) and Bio analyzer 2200 with RIN more than 7.0. Then, approximately 2 *μ*g of total RNA were subjected to isolate the PolyA mRNA using polyT oligo-attached magnetic beads (Invitrogen). After purification, the mRNA was firstly fragmented into small pieces and then reverse-transcribed to create the final cDNA library based on the protocol of the mRNA-Seq sample preparation kit (Illumina, San Diego, USA). The average insert size for the paired-end libraries was about 300 bps. We then performed the paired-end sequencing (100 bps) using the Hiseq3000 platform.

### 2.6. Functional Enrichment Analysis

The sequenced raw data were filtered to remove low-quality tags such as reads with unknown nucleotides “N,” empty reads, and reads with only one copy number. Then, we matched the clean reads to the sequences in the Rattus genome database by Tophat (version 2.0.4) allowing up to two base mismatches. The mapped clean reads were regarded as precise clean reads. For two-factor analysis of variance, we calculated and normalized the number of unambiguous clean reads for each gene to log counts per million using the limma package in R program.

All detected genes were used for the gene ontology (GO) and Kyoto encyclopedia of genes and genomes (KEGG) enrichment analyses. For the GO analysis, a corrected *P* value of <0.05 was considered as the threshold to determine significant enrichment of the gene sets. Similar as GO analysis, a *Q* value ≤0.05 was considered as the threshold to determine significant enrichment of the gene sets for KEGG enrichment analysis.

### 2.7. Quantitative RT-PCR

Total RNA from the two types of cells were isolated using a rapid extraction method (TRI-Reagent, Invitrogen). Real-time PCR was performed on cDNA samples using the FastStart Universal SYBR Green Master (Roche) on Step One Plus system (Applied Biosystems, Foster City, CA, USA). Primers are described in Table S1. The PCR settings used included denaturation (95°C for 2 min) and amplification steps repeated 40 times (95°C for 15 s, 55°C for 30 s, 72°C for 30 s, and acquisition temperature for 15 s). Analysis was conducted using the sequence detection software supplied. For each sample, the delta delta cycle of threshold (ddCt) (crossing point) values were calculated as the Ct of the target gene minus the Ct of the GAPDH gene, assuming PCR efficiency equals to 1. Gene expression was derived according to the equation 2^–ddCt^, and changes in gene expression were expressed relative to levels of the other group.

## 3. Results

### 3.1. The Evaluation of the Cells' Purity

**Figure 1:**
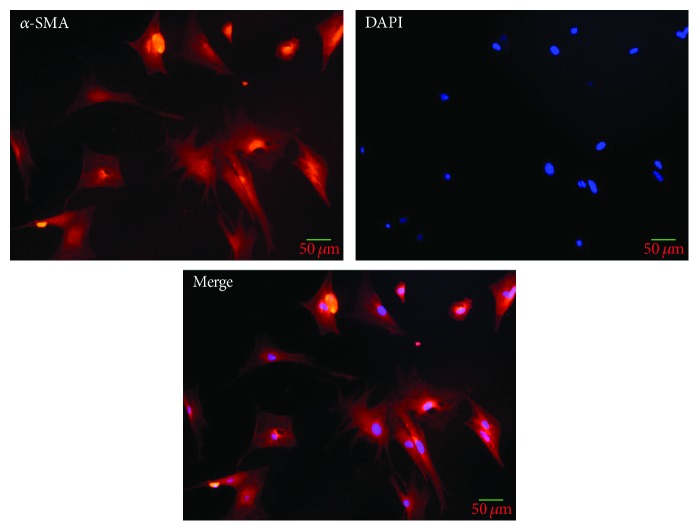
A random view of immunofluorescence visualization of *α*-SMA, a marker for ISCs, was performed. All of the ISCs were *α*-SMA-positive cell.

The immunofluorescence visualization markers for PSCs are known to include *α*-SMA, vimentin, and GFAP. In the previous study, we demonstrated that these markers were also positive in population of ISCs [4]. To identify the purity of the ISCs isolated from islets of the Wistar rats, we used the *α*-SMA staining to confirm that the cells were the right ones we want to analyze by sequencing. As shown in Figure 1, all the cells were positive for *α*-SMA in a random view, indicating that the purity of ISCs was fair.

### 3.2. Evaluation of Gene Expression Profiles

**Figure 2:**
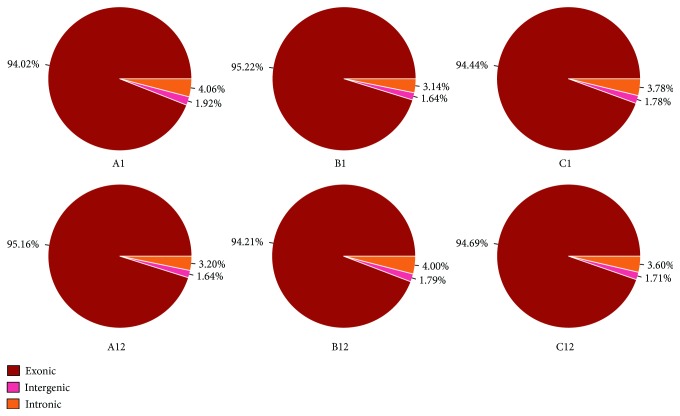
Mapped read distribution of each read.

To verify the sequencing data coverage area and the depth of coverage, Tophat version 2.0.4 was used for analysis. Total numbers of reads generated from each sample ranged from 48,691,180 to 54,536,444. The majority of reads (between 84.66% and 85.18%) were uniquely mapped to the reference Rattus genome sequences across all samples (Table 1). Then, we made statistical analysis on the mapped read distribution from each sample (Figure 2). As shown in Figure 2, the percentage of exonic distribution was ranged from 94.02% to 95.16%. And the intronic distribution was varied from 3.14% to 4.06%. The remaining area was intergenic distributed because of the incompletion of the genetic annotation, which may lead to the detection of new genes or new lncRNA. The total number of detected gene from all the samples was 21,901. Specifically, the number of detected genes from each sample was (A1: 18,603, B1: 18,395, C1: 18,305, A12: 17,984, B12: 18,401, and C12: 18,304).

### 3.3. Analysis of Differentially Expressed Genes

**Figure 3:**
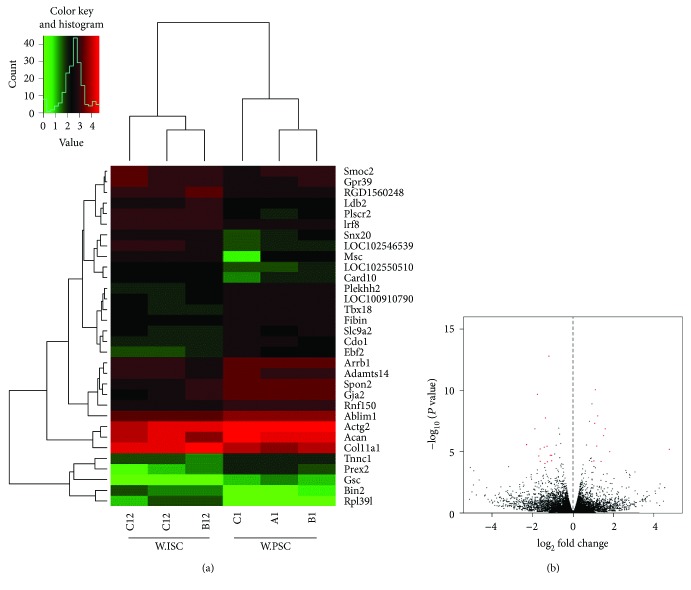
Heatmap and volcano plots showing DEGs between Wistar rats' PSCs and ISCs. Heatmap shows the hierarchically clustered genes (a). Upregulated levels of gene expression are displayed as red bars while downregulated levels are displayed as green bars. Volcano plot shows the overall distribution of DEGs (b). Genes with fold change > 2 and statistical significance are marked with red dots.

To determine the differentially expressed genes (DEG) between PSC and ISC specimens, negative binomial distribution test (NB) was performed. After filtering differentially expressed genes with FDR-adjusted (FDR false discovery rate) *P* value <0.05 and fold change > 2, there were 32 significant differentially expressed genes between PSCs and ISCs. Among them, 14 genes were upregulated and 18 were downregulated in Wistar rats' ISCs (Table 2). The obtained gene expression profiles were visualized in a heatmap and a volcano figure (Figure 3).

### 3.4. The Differential Gene's KEGG Biological Pathway Enrichment Analysis

The biological pathway analysis was referred to Kyoto encyclopedia of genes and genomes (KEGG) database (http://www.genome.jp/). The top 3 most enriched pathways were taurine and hypotaurine metabolism, cysteine and methionine metabolism, and phototransduction (*Q* value <0.05) (Table 3). Cdo1 was both involved in taurine and hypotaurine metabolism and cysteine and methionine metabolism, while Arrb1 was involved in phototransduction pathways.

### 3.5. The Gene Ontology Functional Enrichment Analysis of DEGs

To explore the biological role of the genes modulated in PSCs and ISCs at the transcriptional level, we made function annotation for each DGEs through gene ontology (GO) functional enrichment analysis.

**Figure 4:**
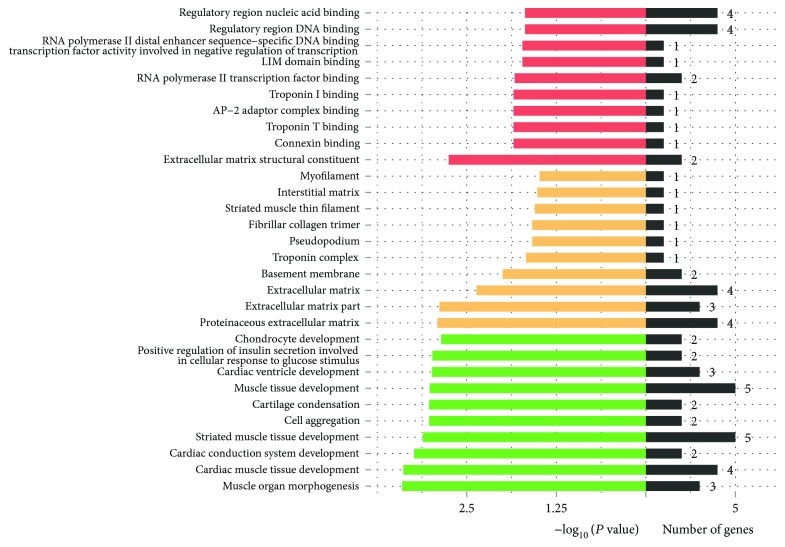
DEGs' GO enrichment classification. Red bars represent the molecular function. Yellow bars represent the cellular component while the green represents the biological process.

The top 10 categories of each part of GO analysis that were significantly enriched with a *Q* value <0.05 are shown in Figure 4.

### 3.6. Validation of mRNA-Seq Results

**Figure 5:**
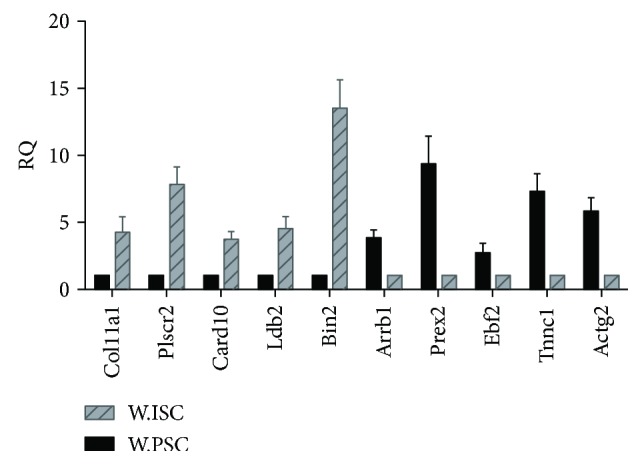
Validation of mRNA-seq to validate the 10 differential expression genes by real-time PCR. Black bars represent the gene expression levels of Wistar rats' PSCs while gray bars represent the relative gene expression levels of Wistar rats' ISCs.

We confirmed the expression level of 10 DEGs (5 upregulated and 5 downregulated genes) using real-time PCR (Figure 5). The genes which we validated should meet the following principles: firstly, the fold change of DEGs was as large as possible. Secondly, the gene expression, at transcription level, was enough to be detected via real-time PCR. Thirdly, the genes were associated with different biological features between Wistar rats' PSCs and ISCs, such as proliferation, migration, and apoptosis.

The results of the validation via real-time PCR showed that, although the RQ was different from the fold change, the tendency was in consistent with the results presented in the transcriptional analysis.

## 4. Discussion

There is the evidence that human fibroblasts exhibit topographic differentiation via genome-wide expression profiling [12]. Fibroblasts from each site displayed distinct and characteristic transcriptional patterns, suggesting that fibroblasts at different locations in the body should be considered distinctly differentiated cell types. The topographic differentiation of fibroblasts results in different biologic specificity. A recent study using immunohistochemistry assay confirmed that there exist *α*-SMA-positive cells in the fibrosis area of islets, which are recognized to be involved in the progression of islet fibrosis [13]. It seems likely that such myofibroblast-like cells in islets should be different from PSCs, since the latter cells are located in the exocrine glands of the pancreas.

Our study showed that there was a difference in transcription levels of Wistar rats' PSCs and ISCs, and there existed 32 differentially expressed genes, accounting for 0.1461% of all the 21,901 genes detected. The mRNA levels of these genes were further confirmed by real-time PCR. The results of the RNA-seq confirmed our hypothesis that ISCs are similar but not identical to PSCs. In this experiment, we focused on the difference at the transcription level between these two types of fibroblasts. So we think that ISCs and PSCs have the same origin but from different locations. PSCs play a part in pancreatic exocrine glands, while ISCs play a part in islets due to their location.

Our data also provided valuable information regarding the subtle but important differences of islet stellate cells versus pancreatic stellate cells. We showed that *COL11A1* expression was significantly upregulated in Wistar rats' ISCs, with a fold change of 2.91.

Minor fibrillar collagens, type V and type XI, were considered to act as nucleators, controlling the assembly of collagen fibrils and participating in proper type II collagen fibril formation [14]. *COL11A1* encodes one of the two chains of collagen type XI, *α*1 chain [15]. *Col11A1* is highly expressed by activated stromal cells in multiple cancers which are mostly invasive, such as oral cavity/pharynx, head and neck, breast, lung, esophagus, stomach, colon, and ovary [16–26]. Recent research has emphasized the role of col11a1 in various cancers. Coll11a1 may play roles in metastasis, angiogenesis, and drug resistance, as well as its potential value in screening tests and as a therapeutic target [27]. One study shows that proCOL11A1 presents a strong immunohistochemical staining within the stroma cells/cancer-associated fibroblasts (CAFs) of pancreatic ductal adenocarcinomas (PDAC), but not strongly costaining with chronic pancreatitis [21]. ProCOL11A1-positive cells presented costaining mesenchymal, stellate, and epithelial markers such as vimentin, *α*-SMA, or desmin in different proportions, suggesting that proCOL11A1-positive cells might be involved in epithelial-mesenchymal transition (EMT).

In agreement with our previous experimental results, this study showed that ISCs contain fewer vitamin A-storing lipid droplets and are more rapidly activated than PSCs in vitro. Activated ISCs express abundant quantity of *α*-SMA and ECM components that may lead to fibrosis. Taking the location of the ISCs into consideration, the activated ISCs are the most likely causes of islet fibrosis in the condition of diabetes. Given our result of mRNA sequencing and considering previous findings, the upregulated expression gene of *Coll11a1* in Wistar rats' ISCs is likely to be a key gene underlying the differential pathophysiology of ISCs and PSCs.

In conclusion, our data showed the differences of ISCs and PSCs at the transcriptional level. Genes like *Coll11a1* may be a key to differences in pathophysiology of ISCs and PSCs. Identification of the gene expression profiles would enrich our current understanding of the ISCs, which also confirmed the previous assumption that ISC and PSC are similar but not identical in morphology and phenotype. In our previous study, comparing with normal status, ISCs in diabetic status showed a significantly greater migration, a larger rate of apoptosis and viability, and a higher level of ECM component secretion [28]. Provided that there existed a molecular switch that can be used to reverse this phenomenon and subsequently relieve the islet fibrosis in terminal stage of diabetes, this could be a cue to develop novel efficiency therapy approaches for diabetes.

## Figures and Tables

**Table 1:** Comparisons between clean data and Rattus genome sequences.

Mapped statistics	A1	B1	C1	A12	B12	C12
Total reads	48,691,180 (100%)	53,917,892 (100%)	51,532,204 (100%)	50,777,686 (100%)	53,749,152 (100%)	54,5 36,444 (100%)
Total mapped	44,505,452 (91.4%)	49,095,515 (91.06%)	46,914,556 (91.04%)	46,188,430 (90.96%)	49,072,733 (91.3%)	49,552,463 (90.86%)
Multiple mapped	3,128,881 (6.43%)	3,429,714 (6.36%)	3,288,274 (6.38%)	3,172,290 (6.25%)	3,288,591 (6.12%)	3,290,626 (6.03%)
Uniquely mapped	41,376,571 (84.98%)	45,665,801 (84.7%)	43,626,282 (84.66%)	43,016,140 (84.71%)	45,784,142 (85.18%)	46,261,837 (84.83%)

**Table 2:** The significant differentially expressed genes between PSCs and ISCs.

Genes	Fold change	*Q* value
Upregulated genes in Wistar rats' ISCs		
Rpl39l	77.34	0.014
Bin2	26.93	0.01
LOC102546539	7.10	1.00*E* − 21
Plscr2	3.76	8.13*E* − 15
Msc	3.49	0.01
Snx20	2.99	0.00
Col11a1	2.91	2.75*E* − 21
LOC102550510	2.84	0.00
Card10	2.59	0.03
Ldb2	2.32	2.65*E* − 05
Gpr39	2.23	0.00
RGD1560248	2.13	2.75*E* − 07
Irf8	2.09	8.20*E* − 05
Smoc2	2.03	0.03
Downregulated genes in Wistar rats' ISCs		
Rnf150	0.48	0.01
Adamts14	0.48	0.03
Arrb1	0.46	0.01
Spon2	0.44	5.93*E* − 10
Fibin	0.42	0.03
Ablim1	0.41	0.00
LOC100910790	0.39	3.53*E* − 05
Acan	0.38	0.04
Slc9a2	0.37	0.00
Actg2	0.32	0.03
Cdo1	0.32	0.00
Tnnc1	0.31	0.01
Plekhh2	0.29	5.72*E* − 07
Ebf2	0.27	0.00
Gja5	0.23	7.12*E* − 17
Tbx18	0.228723292	1.15*E* − 16
Prex2	0.203772172	0.002737756
Gsc	0	0.000404614

**Table 3:** KEGG pathway analysis of DEGs in Wistar rats' PSCs and ISCs.

Term	ID	*Q* value	Gene ID	Gene
Taurine and hypotaurine metabolism	rno00430	0.008177	81,718	Cdo1
Phototransduction	rno04744	0.025229	25,387	Arrb1
Cysteine and methionine metabolism	rno00270	0.036734	81,718	Cdo1

## Supplementary Materials

Supplementary Materials Table S1: the primers of validated genes.
