# Supplementary material for: A Transcriptional Sequencing Analysis of Islet Stellate Cell and Pancreatic Stellate Cell
Source: J Diabetes Res. 2018 Jan 24;2018:7361684. doi: 10.1155/2018/7361684 (PMC5830286; doi:10.1155/2018/7361684)
Supplement: Supplementary Materials — Table S1: the primers of validated genes. [file 7361684.f1.pdf]

Table S1 The primers of validated genes.

Gene	Forward primer	Reverse primer
Col11a1	CTATTGGTTCAGTTGGTGGT	CCTTTCTCTCCTCTCTCTCC
Plscr2	ACTCAACTCACTTGGAGCCG	TTGGAAAGCTGCTGGTGGAT
Card10	CTCAGAGCTAGAGCGAAGCC	AGATTGCGGATAAGGCGAGG
Ldb2	CAGCAAGAAGAAGACCACA	CAAACTCACCTCCCATCA
Bin2	GAGCAAACTGGAGAAGCA	GGGGAGAGATGACTAAGGAG
Arrb1	TTGTGTTTGAGGACTTTGCT	CTATCTGTTGTTGAGGTGTGG
Prex2	ACTGGAGCAAGCCATCACTC	AGGAGTCCGGTCTCTAACCC
Ebf2	ATGTGCTGGCTGTTTCTG	CTTGGACTGATGGCTTTG
Tnnc1	CAAAGGGAAGTCTGAGGAG	CAGTTCATCCAAGTCAATGTAG
Actg2	TAAAGCCAACAGGGAGAAG	GCATAGAGCGAGAGCACA
Gapdh	TCTCTGCTCCTCCCTGTTC	ACACCGACCTTCACCATCT
